# A new approach to urinalysis: effectiveness of a contingency management program among adolescent offenders in Spain

**DOI:** 10.3389/fpsyg.2024.1364967

**Published:** 2024-05-03

**Authors:** Álvaro Fernández-Moreno, David Roncero, Román D. Moreno-Fernández

**Affiliations:** Faculty of Education and Psychology, Universidad Francisco de Vitoria, Madrid, Spain

**Keywords:** urinalysis, drug consumption, treatment, effectiveness, juvenile delinquency, contingency management program

## Abstract

**Background:**

When addressing antisocial behaviour among adolescents, programs based on the paradigm of positive psychology through enhancing self-efficacy have demonstrated their effectiveness in furthering the positive development of young people with a history of antisocial behaviour. Nevertheless, there has been little research into the effectiveness of these type of programs in mitigating substance abuse among juvenile offenders. The aim of this paper is to analyse the effectiveness of a contingency management program in reducing the prevalence of relapses into drug consumption among adolescents who have committed serious crimes.

**Methods:**

The study consisted of a sample of 91 male adolescents, between 15 and 19 years, in juvenile detention, who were divided into two treatment groups. For both groups, biological testing was used to detect drug consumption upon their re-turn from leave permits from the Centre.

**Results:**

The quasi-experimental group had significantly lower rates of relapse than the quasi-control group. Furthermore, being part of the quasi-experimental group was a significant predictor of reduced rates of relapses.

**Conclusion:**

The results suggest that the incorporation of treatment strategies which reinforce feelings of self-efficacy and adequate orientation towards the future, as a complement to disciplinary sanctions, are effective in reducing relapses in drug use among adolescent offenders.

## 1 Introduction

Drug consumption has been extensively studied as one of the primary risk factors associated with juvenile delinquency (López and Rodríguez-Arias, 2012; Bonta and Andrews, 2017; Aebi et al., 2021; Hiller et al., 2021). A high prevalence of severe drug consumption is evident among youths involved in serious crimes (Pérez and Ruiz, 2017), as well as among those with a history of early and persistent criminal behaviour (Kerridge et al., 2020; Brislin et al., 2021).

In the United States, there is a long tradition of early treatment of problems associated with drug addiction among adolescent offenders through outpatient programs or interventions during detention. Since the 1990’s, Juvenile Drug Treatment Courts have existed in the United States to deal with youth drug addiction (Ledgerwood and Cunningham, 2019). Although there is considerable scientific literature on these types of intervention programs, a number of questions remain unsolved, especially regarding the effectiveness of these programs (Ali et al., 2022). Research suggests that a number of intervention models have proven successful in treating drug consumption among adolescent offenders, such as the Systemic Family Therapy, Multidimensional Family Therapy, Cognitive-Behavioural Therapy and Brief Motivational Interviewing (Tripodi et al., 2010; Tripodi and Bender, 2011). Similarly, Dopp et al. (2017) affirm that family-based treatment programs can successfully mitigate the social and economic consequences of crime committed by adolescents. Despite these findings, research comparing the effectiveness of the different intervention models have not found statistically significant differences between them, without any particular model showing greater levels of effectiveness compared to the rest (Tanner-Smith et al., 2016; Hiller et al., 2021).

One strategy which has proven to be an effective compliment to well-consolidated treatment programs for substance abuse is the contingency management program dealing with positives in urine drug screening (Johnson et al., 2019; López-Pelayo et al., 2020). This program is particularly effective with adolescent offenders (Henggeler et al., 2012). In fact, given the lack of reliability of self-report questionnaires by adolescent offenders in treatment for substance abuse about their drug consumption (Dembo et al., 2022), it is increasingly common for studies to turn to urine drug screening to analyse substance abuse among offenders (Demir et al., 2020). Additionally, in recent decades outpatient therapeutic models have become increasingly important in addressing antisocial behaviour and substance abuse (Delen et al., 2021), particularly contingency management programs.

In Spain, Organic Law 5/2000, January 12, on the criminal responsibility of minors, refers to the need to apply treatment programs which offer the greatest possibilities of success. While there is no independent tribunal in the Spanish legal system, for years, Juvenile Courts have been applying therapeutic programs for the treatment of substance abuse, either through outpatient programs or in detention (Lázaro-Pérez, 2001). Thus, the courts are clearly aware of the link between criminal behaviour and drug abuse (Bujosa Vadell et al., 2021), the most prevalent being cannabis (San Juan et al., 2009; Contreras et al., 2012; Uceda-Maza et al., 2016; Vega-Cauich and Zumárraga-García, 2019). An exhaustive search revealed no studies of the Spanish population which measure the effectiveness of the aforementioned intervention or contingency management therapies in outpatient programs. There is also a striking lack of research into both the effectiveness of these programs in detention, either in open, semi-open or closed regimes, or into the patterns of substance abuse among adolescent offenders.

In response to this situation, Fernández-Moreno et al. (2022) developed an intervention program for the treatment of substance abuse among young offenders with severe drug abuse problems. They found that adapting cognitive-behavioural techniques, with an orientation towards the future, applied through the prism of positive psychology, produced a statistically significant reduction in problems associated with substance abuse, measured using the T-ASI (Díaz and Castro-Fornieles, 2008), with a high effect size (*η*^2^ = 0.55) and proving more effective than the active control group using only individual cognitive-behavioural therapy (*η*^2^ = 0.16). This program is one of the few intervention models which address antisocial behaviour from the paradigm of positive psychology despite the fact that these have proven effective in the reduction of antisocial behaviour (Riffo-Allende, 2021). Positive psychology is a general paradigm which reorients the perspective of research, prevention and clinical practice towards general strengths and resources (Carrea and Mandil, 2011; Fernández-Ríos and Vilariño Vázquez, 2018). Intervention programs explicitly based on positive psychology are oriented towards the promotion of positive emotions, reinforcing the resources and experiences of wellbeing (Santamaría-Cárdaba, 2018; Toribio et al., 2018). Studies into positive psychology have found that promoting a context of wellbeing during adolescence reduces the likelihood of the appearance of psychopathologies (Bohlmeijer et al., 2017). A fundamental strategy of interventions based on positive psychology is to foster an orientation towards the future, which has proven to be an important factor in the positive development, even for young people with a long history of antisocial behaviour and severe substance abuse problems (Brooks et al., 2018).

In light of the above, and with the aim of further exploring the findings of Fernández-Moreno et al. (2022), this study proposes two primary research aims: first, to identify the pattern of drug abuse among adolescent offenders in juvenile detention through the use of urine drug screening; second, to evaluate the effectiveness of interventions using contingency management method with biological drug testing and using techniques of positive psychology for minor offenders with high consumption of cannabis. This type of intervention has been conducted at the CEMJTC (Centro de Ejecución de Medidas Judiciales Teresa de Calcuta) in the Community of Madrid.

## 2 Methods

### 2.1 Participants

The initial sample for the study consisted of 149 inmates of the CEMJTC, 145 males and 4 females, serving a custodial sentence in closed or semi-open regime between January 1st, 2017 and January 1st, 2020 and benefitting from a permit allowing them to leave the Centre for education, employment or recreation without accompaniment. At the start of their detention period, participants were from 15 to 19 years of age, with a mean age of 16.79 ± SEM = 0.129 years. Regarding nationality, 40.43% of participants were Spanish, 28.36% from Latin America, 28.36% from the Maghreb and 4.96% from other European countries. According to official records of the centre, the majority of the adolescents came from dysfunctional family backgrounds (61.6%). Economic issues were the most prevalent problem within these families (47.5%), followed by relational difficulties (30.2%), multi-faceted family issues (29.2%), involvement in delinquent behaviour (22.7%), and substance abuse (16.2%) (Agencia para la Reeducación y Reinserción del Menor Infractor [ARRMI], 2021). Most of the crimes committed by the participants were of a violent nature, primarily targeting property (81.5%). Prior to their incarceration, 33.7% of the adolescents in this centre had served previous jail sentences, and 65.2% had a history of multiple criminal offenses (Agencia para la Reeducación y Reinserción del Menor Infractor [ARRMI], 2021).

**Figure 1:**
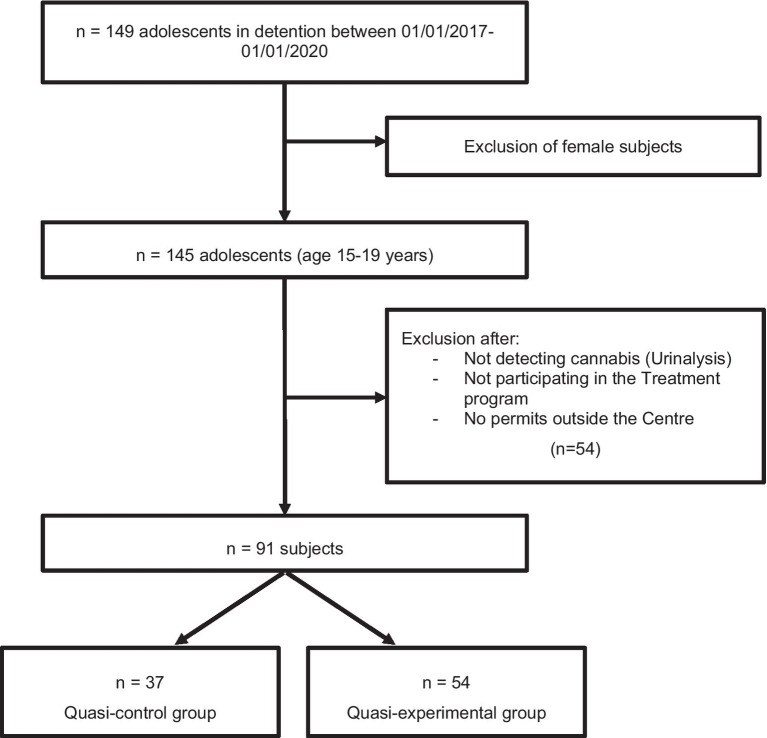
Flowchart of the sample selection process.

From this initial sample, participants were selected on the basis of the following inclusion criteria (Figure 1):

- Male, given that the proportion of females in the initial sample was very low. Furthermore, the majority of juvenile delinquents in detention are males, some 85.6%, in the Community of Madrid in the year 2020 (Agencia para la Reeducación y Reinserción del Menor Infractor [ARRMI], 2021).
- Participation in the Drug Abuse Treatment program at the CEMJTC (Fernández-Moreno et al., 2022). Those included in this program have been identified by the therapy team of the Centre as having severe substance abuse problems requiring specific treatment.
- Cannabis consumption prior to internment in the Centre, detected through a urine drug test (UDT or urinalysis).
- To have benefitted from a permit allowing them to leave the Centre without accompaniment.

With the application of this inclusion criteria, the definitive sample consisted of 91 participants, of whom 57 were assigned to the quasi-experimental group and 34 to the quasi-control group.

### 2.2 Instruments

#### 2.2.1 Biological tests to detect drug consumption

In order to detect the consumption of opiates, cocaine, cannabis, ecstasy, amphetamines, MDMA and benzodiazepines, urine drug screening tests (UDT or urinalysis) were conducted both at the start and during detention (Urine Rapid Test Dipstick, THC-50, COC-300, BZO-300, MTD-300, AMP-1000, MOP-300; Hangzhou Alltest Biotech Co. Ltd.). Alcohol consumption was tested using a breathalyser after release permits during detention. Alcohol consumption could not be measured at the start of detention since the adolescents spent at least 24 h in police custody upon their detention.

Analytic drug testing was performed following these basic steps:

- The adolescent must provide their written consent prior to conducting the drug test.
- If the adolescent refuses to give their consent the drug tests are not conducted. In this case, if there is any indication of intoxication, the adolescent is attended by the Medical Staff of the Centre, who make their evaluation and the corresponding medical report.
- The Medical Staff of the Centre collect urine samples and a breathalyser test.

**Figure 2:**
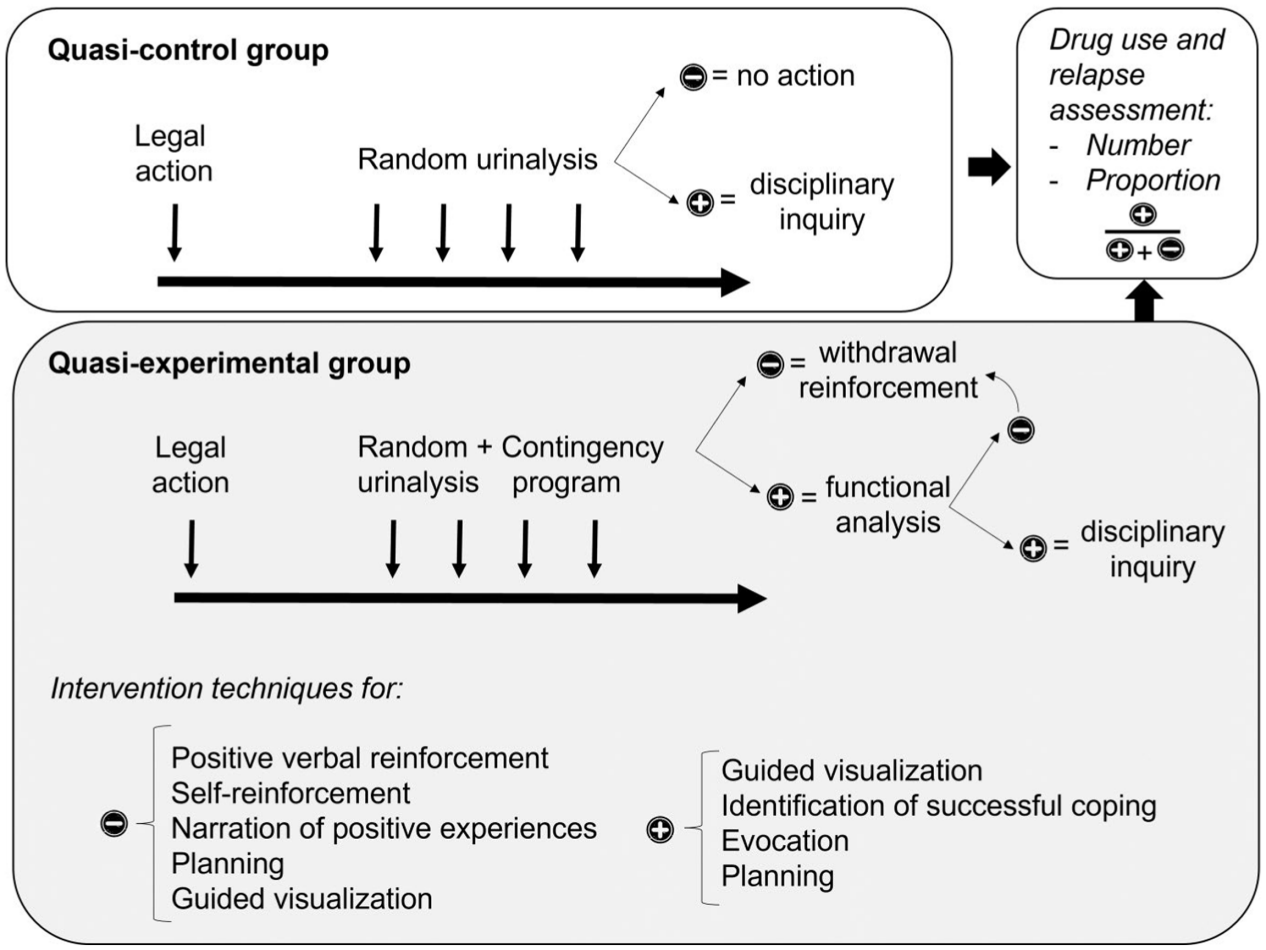
Flow-chart of the design of the study. (−): negative result (no drug consumption); (+) positive result (relapse).

A total of 829 biological tests were performed to detect drug use during the course of this study, 91 at the start of the detention period and 738 follow-up tests. Participants did not take the same number of tests. The number of tests varied (8.11 ± 0.859) de-pending on the number of permits the participants benefitted from during their detention period, determined by judicial and technical criteria. Thus, two different measures were used to calculate relapses into drug use. First, the number of participants with any detected drug consumption during their internment compared to the number of those who remained abstinent. Second, the number of positive drug tests divided by the total number of tests for each participant for each of the drugs being analysed (Figure 2).

#### 2.2.2 Consultation of criminal record

The criminal record of each participant in the juvenile Centre was consulted for sociodemographic information: age, gender and nationality.

### 2.3 Procedure

This research project used a quasi-experimental design with a quasi-control group drawn from a previous cohort. The participants were separated into two groups for different treatment conditions. The quasi-experimental group consisted of 57 participants who met the inclusion criteria of the study and benefitted from release permits from the CEMJTC between January 1st, 2018 and January 1st, 2020. The quasi-control group consisted of 34 adolescents who met the inclusion criteria of the study and benefitted from release permits from the CEMJTC between January 1st, 2017 and December 31st, 2017.

The quasi-control group was created from adolescents from a prior time period for various reasons; firstly, the internal procedures of the CEMJTC made it necessary to apply the intervention program simultaneously to all minors in the Centre; secondly, the educational and technical personnel of the Centre, and all other treatment programs, remained the same, without significant changes during both time periods included in the research. Thus, rather than took for a quasi-control group in another Centre, with different procedures, personnel and treatment programs, priority was given to maintaining the same context for the quasi-experiment and quasi-control groups.

Biological drug tests were conducted with both groups to detect drug consumption (cannabis, opiates, benzodiazepines, cocaine, MDMA, Methamphetamine and alcohol) upon their return to the Centre after leave permits for education, employment or recreation, either ordinary or extraordinary.

#### 2.3.1 Active quasi-control group

For this group, in addition to the therapy provided at the CEMJTC (Fernández-Moreno et al., 2022), random drug testing protocol was conducted after their return to the Centre after leave permits for education, employment or recreation. No actions were taken in the event of negative results (no consumption); in the case of a positive result (repeat of drug consumption) a disciplinary report was created, part of the general action protocol for the Centre.

#### 2.3.2 Quasi-experimental group

For this group, in addition to the therapy provided by the CEMJTC (Fernández-Moreno et al., 2022), a new drug testing protocol was introduced which emphasised positive reinforcement in maintaining abstinence and early intervention in the event of relapses using a series of actions:

1. Weekly reporting to the Technical Team, psychologist or social worker, of the results of drug testing for use during interventions with participants.
2. Positive reinforcement in maintaining abstinence, supervising the responses of participants in the case of situations that revive habits of drug consumption. This step includes the review and adjustment of contingency plans part of the Treatment program. Specifically, the interventions carried out included techniques of positive verbal reinforcement, self-reinforcement, narration of positive experiences during the leave permit, planning for the next leave permit, and guided visualization of a future without drug consumption.
3. Early intervention in the event of relapses through the functional analysis of the circumstances of renewed consumption, identification of triggers, problematic behaviour and its consequences. In this step the work of the Treatment program is resumed, re-evaluating the Stages of Change proposed by DiClemente and Prochaska (1982). In the case of a relapse, a positive drug test, it was decided not to immediately revoke the privileges of the participant but rather to give them the opportunity address the relapse in a therapeutic manner and return to abstinence. For this, techniques were applied designed to foster a positive orientation towards the future (Brooks et al., 2018), which may be defined as the presence of realistic aspirations, adequate expectations and the development of planning skills. The specific interventions used included guided visualization of a future without drug consumption, identification of past successful coping situations, evocation of internal motivations to maintain abstinence, and planning for the next leave permit.
4. If the participant does not respond adequately to the intervention and persists in their drug consumption, the disciplinary regime of the Centre is applied, as an integral element of therapeutic interventions in the context of detention.

These actions are based on the positive psychology paradigm (Santamaría-Cárdaba, 2018; Toribio et al., 2018), seeking to foment positive emotions during processes of change, reinforcing the resources and experiences of wellbeing through the consolidation of positive feelings of self-efficacy. In this sense, previous evidence on other addictions during adolescence revealed positive results when addressing self-efficacy (Gullo et al., 2010; Durkin et al., 2021; Favini et al., 2023).

### 2.4 Data analysis

First, a descriptive analysis was made of the drug consumption of participants detected at the start of their detention. The equivalence of the quasi-experimental and quasi-control groups was then verified using a Chi squared test, comparing the drug consumption of both groups at the start of their detention period.

A Chi squared test (*χ*^2^) was performed to determine the frequency participants had a relapse in drug use during their detention period according to the different groups.

The Student’s *t* test was used to analyse the differences in proportion of relapses according to the different drugs consumed during detention among both groups. The effect size was calculated using Cohen’s d.

Finally, a multiple linear regression test was determined including the following variables: proportion of relapses in any drug consumption and proportion of relapses in consuming cannabis in order to determine the possible association between a dynamic variable, the participation in the contingency management program, and two static variables, history of drug use (specifically benzodiazepines, cocaine and amphetamines/MDMA).

Data was analysed using the SPSS statistics suite, version 21.

## 3 Results

Drug testing using urine drug screening showed, at the start of their detention, some type of drug consumption among 81.2% of the 149 participants in the study. The most frequently detected substance was cannabis (80.5%), followed by benzodiazepines (23.5%), cocaine (14.1%), amphetamines/MDMA (2%) and opiates (0.7%).

**Figure 3:**
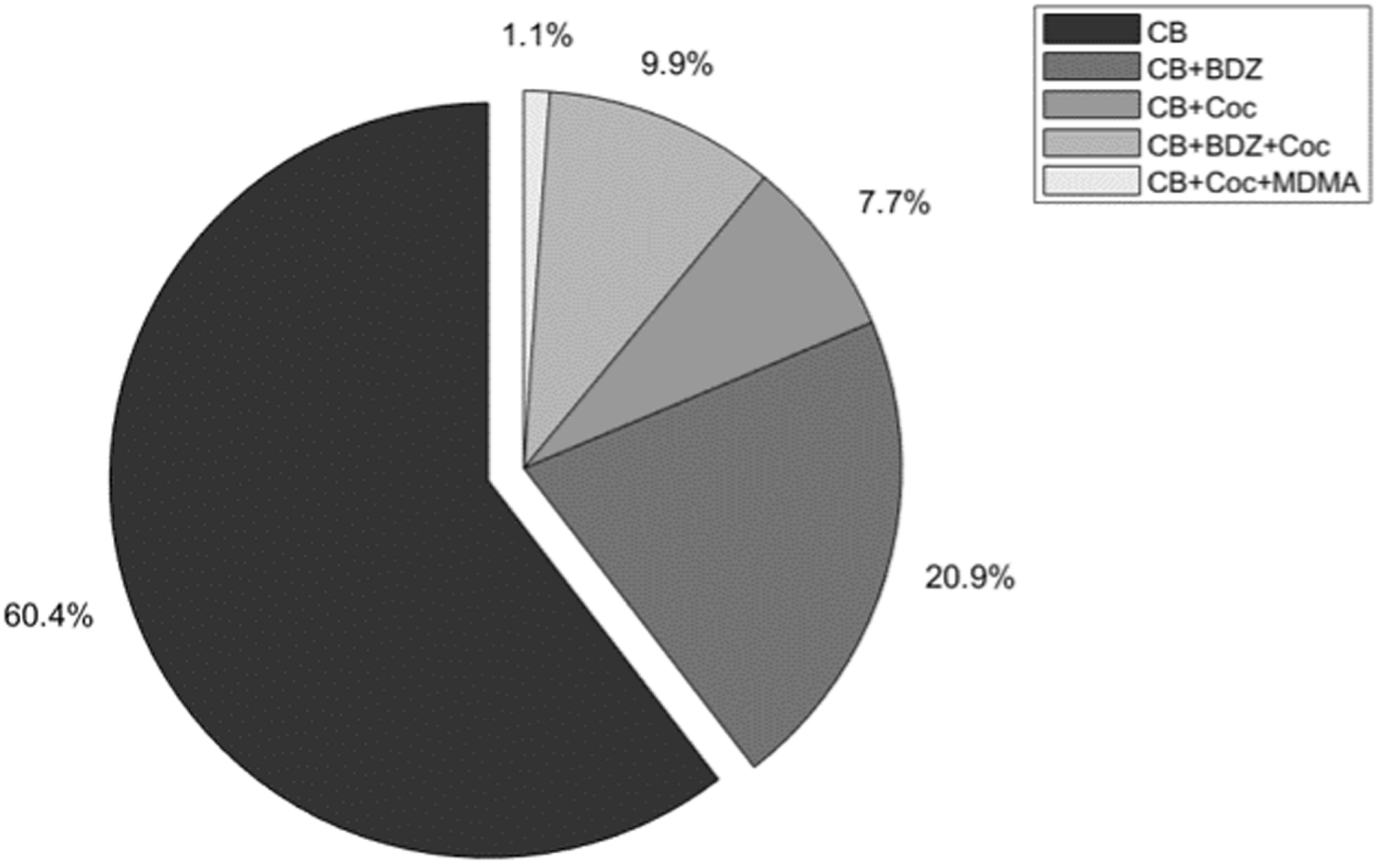
Results of biological testing for drug consumption at the start of detention. *N* = 91; BDZ, Benzodiazepines; CB, Cannabis; Coc, Cocaine.

With the application of the inclusion criteria, the final sample consisted of 91 participants. Drug testing at the start of detention showed the following: 60.4% tested positive for cannabis consumption; the combination of cannabis – benzodiazepines appeared in 20.9% of cases; the combination cannabis – benzodiazepines – cocaine in 19.9%; cannabis – cocaine in 7.7%; and the combination cannabis – cocaine – MDMA was detected in only one case (Figure 3).

Biological testing showed that the substances most frequently accompanying the consumption of cannabis at the start of detention were benzodiazepines and cocaine, detected among 30.8 and 18.7% of participants, respectively. The homogeneity between the quasi-experimental and quasi-control groups was verified in terms of drug consumption at the start of detention, with no significant differences between the two groups (*χ*^2^ *=* 3.498, *p = 0.*478).

The percentage of participants consuming drugs during their leave permits was analysed. As indicated in Table 1, the results of drug screening show that 48.4% of participants maintained abstinence during their detention period, while 51.6% consumed some type of drug during leave permits, principally cannabis (41.8%), with significantly less frequent consumption of benzodiazepines (11%) and cocaine (8.8%).

**Table 1:** Percentage of participants who consumed drugs during the detention period.

Type of drug	Total sample (*n* = 91)	Quasi-control Group (*n* = 34)	Quasi-experimental Group (*n* = 57)	*χ*^2^	*p*
	*n* (%)	*n* (%)	*n* (%)		
Any substance	47 (51.6)	18 (52.9)	29 (50.9)	0.036	0.849
Cannabis	38 (41.8)	17 (50.0)	21 (36.8)	1.516	0.218
Benzodiazepines	10 (11.0)	2 (5.9)	8 (14.0)	1.447	0.229
Cocaine	8 (8.8)	2 (5.9)	6 (10.5)	0.573	0.449
Alcohol	7 (7.7)	2 (5.9)	5 (8.8)	0.250	0.617
Other substances	1 (1.1)	0 (0)	1 (1.8)	0.603	0.437

To determine the effectiveness of the contingency management program, the study determined the differences between the quasi-experimental and quasi-control groups in terms of the percentage of participants who suffered a relapse during detention. No significant differences were found, indicating that the number of participants who consumed drugs during their detention period was similar in both groups (Table 1).

The study then analysed if proportion of relapses into drug consumption during detention varied according to participation in the contingency management program.

**Figure 4:**
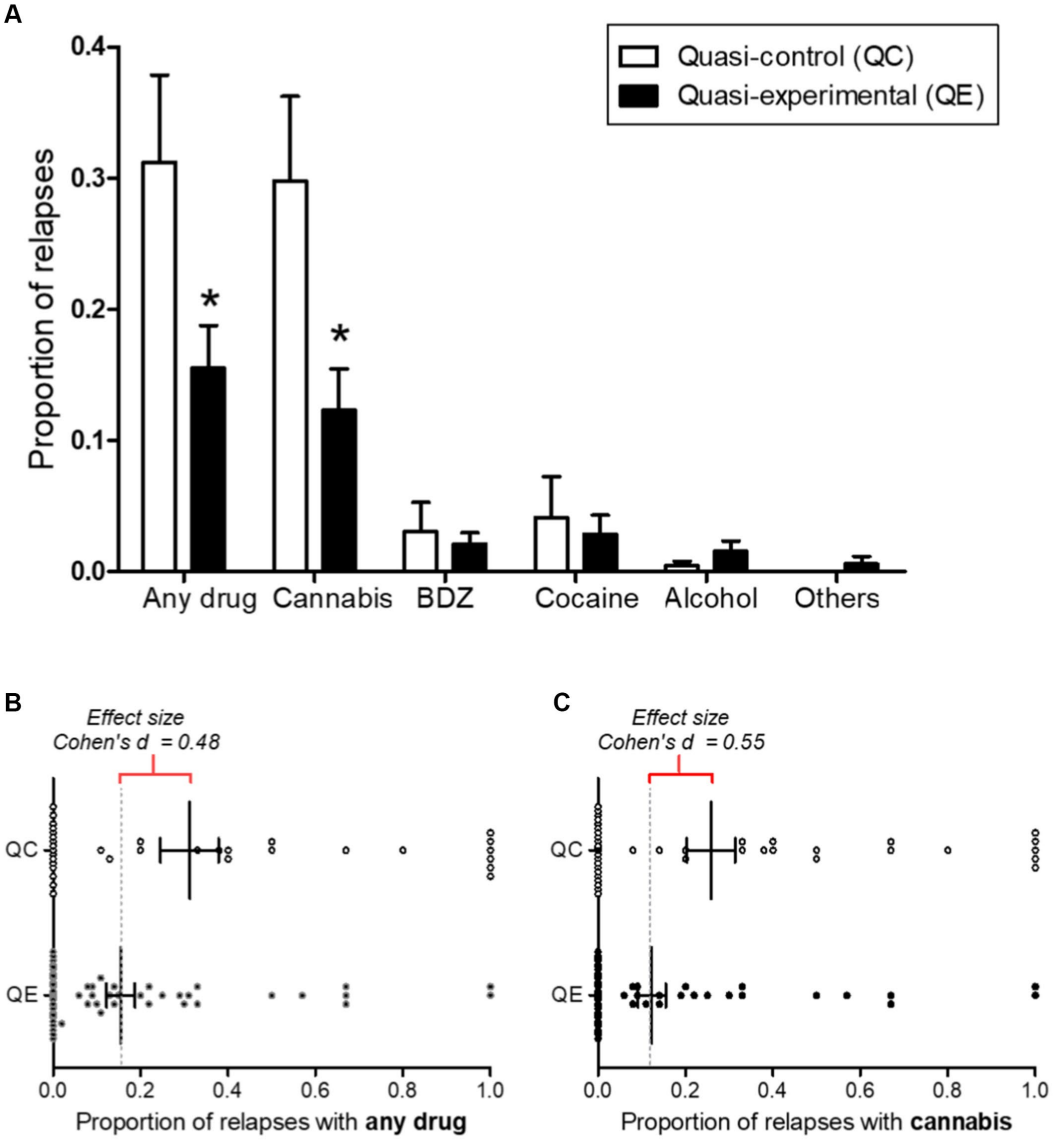
**(A)** Proportion of relapses in drug consumption according to participation in the contingency management program. Error bars represent s.e.m. BDZ, benzodiazepines; QC, Quasi-control (*n* = 34); QE, Quasi-experimental (*n* = 57); *, *p* < 0.05. **(B)** Effect size of proportion of relapses with any drug. **(C)** Effect size of proportion of relapses with cannabis.

As shown in Figure 4, in this case there were significant differences in the consumption of any type of drug, with the quasi-control showing a higher proportion of relapses than the quasi-experimental group (Figure 4A; *t*_(89)_ = 2.123; *p* = 0.039), with a medium effect size (Figure 4B; *d = 0*.48). Significant differences were also found in the consumption of cannabis, with the quasi-control group showing higher levels of consumption than the quasi-experimental group (Figure 4C; *t*_(89)_ = 2.431; *p* = 0.019), also with a medium effect size (Figure 4C; *d* = 0.55). For other drugs, the proportion of consumption was very low in both groups with no significant differences between them (Figure 4A).

Finally, a multiple linear regression analysis was conducted for the variable proportion of relapses in the consumption of any drug and the proportion of relapse in the consumption of cannabis. In both cases, the predictive variables were participation in the contingency management program, and consumption prior to detention of benzodiazepines, cocaine and amphetamines/MDMA. The two regression models were significant, both in terms of relapses in the consumption of any drug (*F =* 2.582, *p = 0.*043), and in relapses in the consumption of cannabis (*F =* 2.800, *p = 0.*031). Table 2 shows that participation in the contingency management program is negatively associated with relapses in the consumption of any type of drug (*B =* −0.147, *p = 0.*031) and with relapse in cannabis consumption (*B =* −0.174, *p = 0.*009). However, consumption of benzodiazepines, cocaine and amphetamines/MDMA prior to detention did not show any significant association with relapses in drug consumption during detention.

**Table 2:** Predictive model of positive testing for any substance and cannabis.

	B	SE	*p*
*Relapse with any drug*			
Participation in the contingency management program	−0.147	0.060	0.031
Prior consumption of benzodiazepines	0.061	0.071	0.395
Prior consumption of cocaine	0.138	0.087	0.118
Prior consumption of amphetamines/MDMA	−0.291	0.317	0.361
*Relapse with cannabis*			
Participation in the contingency management program	−0.174	0.058	0.009
Prior consumption of benzodiazepines	0.087	0.070	0.213
Prior consumption of cocaine	0.062	0.085	0.469
Prior consumption of amphetamines/MDMA	−0.325	0.309	0.297

## 4 Discussion

The research objectives of this study were to identify the pattern of drug consumption among adolescent offenders in juvenile detention and to evaluate the effectiveness of interventions using a contingency protocol using techniques of positive psychology to reduce the frequency of relapses into substance abuse.

The study made use of urine drug screening tests (UDT or urinalysis) to detect drug consumption both at the start of the detention period and in determining relapses into drug use relapses during leave permits from the Centre. The use of biological testing has become increasing prevalent for the detection of drug use and the evaluation of the effectiveness of intervention programs (Demir et al., 2020). Urine drug screening tests (UDT or urinalysis) offer a quick, low-cost tool that is very accurate and effective in detecting drug use when used with the proper controls. UDT offers advantages over other common techniques, such as self-reporting, which may be unreliable (Dembo et al., 2022). Urinalysis is particularly valuable for use with those in judicial detention, such as the participants of this study, as drug consumption during leave permits can negatively impact their legal situation. Thus, biological testing, such as urine drug screening tests (UDT or urinalysis), is not only an effective tool to analyse drug consumption and monitoring but also facilitates the orientation and application of treatment programs in detention aimed at reducing the prevalence of relapses.

Urine drug screening tests (UDT or urinalysis) conducted at the start of detention showed highly problematic patterns of consumption, in line with the results of previous research (San Juan et al., 2009; Uceda-Maza et al., 2016; Vega-Cauich and Zumárraga-García, 2019), confirming that substance abuse is a common problem in the penitentiary population in general and specifically among young offenders (Indig et al., 2016; Calero-Plaza et al., 2020). Although inmates entered the CEMJTC some one to three days after detention, some form of drug consumption was detected in four out of five inmates. The most frequently detected substance was cannabis, for which virtually all participants tested positive. Significant differences were found in the consumption of cannabis compared to other substances, such as benzodiazepines and cocaine, and especially amphetamines/MDMA and opiates. The results of this study revealed a high degree of poli-consumption (39.6%), most commonly, in addition to cannabis, the consumption of benzodiazepines and cocaine. This is particularly important given that poli-consumption is generally associated with greater degrees of social dysfunction and even higher rates of criminal recidivism (Davis et al., 2020).

With regards to relapses, cannabis was found to be the most commonly detected drug both prior to their detention period and upon the return of participants to the Centre after leave permits. The results of the study show how cannabis is currently the most prevalent drug in substance abuse problems among young offenders in Spain (Vega-Cauich and Zumárraga-García, 2019; Calero-Plaza et al., 2020).

The results of the study show that the contingency management program is not more effective than the usual drug treatment programs of the CEMJTC when considering only the number of participants who are entirely abstinent. This is in line with the findings of previous studies (Kaminer et al., 2014; Johnson et al., 2019). For example, the team of Johnson et al. (2019) applied a program of contingency management over 18 months to a population of young people with problems of cannabis use and a history of psychosis. The study found no significant reduction in relapses detected using urine drug screening. However, our study with young offenders found that the contingency management program positively contributed to a reduction in relapses into cannabis use during juvenile detention; that is, relapses were less frequent among participants with whom the contingency management program was applied. These results are in line with those of Fernández-Moreno et al. (2022), who found evidence of the effectiveness of interventions based on cognitive-behavioural models applied using a paradigm of positive psychology to reduce the problems associated with alcohol and drug consumption among juvenile offenders. These results are particularly noteworthy given the limited effectiveness of intervention programs aiming to address substance abuse, including cannabis (Tanner-Smith et al., 2016).

Nevertheless, participation in the contingency management program emerged as a significant predictor of a reduction in relapses compared to other variables associated with substance abuse. This highlights the importance of dynamic and manipulable factors related to treatment as opposed to other factors of a historical or static nature which are traditionally associated with the greater probability of relapse into renewed drug use (Nguyen et al., 2020).

The results of the study raise a number of considerations for the treatment of substance abuse among juvenile offenders. Firstly, the contingency management program for the results of urine drug screening and breathalysers are tools that may be used as complementary strategies to psycho-therapeutic interventions. It appears that the application of protocols for the contingency management programs that support psycho-therapeutic interventions oriented towards the treatment of substance abuse, especially cannabis, can enhance their effectiveness (Henggeler et al., 2012). The use of techniques aimed at fostering an orientation towards the future, based on the paradigm of positive psychology, may be effective in preventing relapses into drug use and help strengthen the commitment to therapy and abstinence (Brooks et al., 2018).

In light of these findings, it is important to emphasise the importance of positive reinforcement in promoting abstinence and dealing quickly with relapses through actions based on the positive psychology paradigm (Santamaría-Cárdaba, 2018; Toribio et al., 2018), seeking to foment positive emotions during processes of change, reinforcing the resources and experiences of wellbeing through the consolidation of positive feelings of self-efficacy.

These interventions can help foster realistic aspirations, adequate expectations and the development of planning skills among participants. In the event of relapses into drug consumption, it is important for the inmate to address the relapse in a therapeutic manner and return to abstinence. These actions may contribute to greater engagement with therapists and interiorisation of the therapeutic process. In order to validate the results of this study, it would be interesting to apply and replicate the intervention program into other centres and to control for nationality and cultural diversity.

However, any analysis of these results must take into consideration the limitations of a study of this type.

Firstly, the quasi-control group was selected from a cohort previous to that of the experimental group. This selection of the quasi-control group was due to the difficulty in finding a simultaneous group with similar characteristics as the treatment group. In fact, the information and characterization of the participants is limited given that the intervention was conducted in a professional context oriented to therapeutic processes, but not planned beforehand as research. Nevertheless, the groups were homogeneous in terms of their prior drug consumption habits. Furthermore, there were contextual factors that support this choice of control group; they had the same treatment programs with the exception of the contingency management program, which would not have been possible using a quasi-control group from another centre.

Secondly, the program was applied in a single centre using a relatively small and exclusively male sample. For more solid conclusions, it is recommended that future research include larger and more diverse samples.

The fact that the effectiveness of the program was evaluated using biological testing is one of the strengths of the present study, offering reliable evidence of the prevalence of relapses into drug use. However, given the lack of any follow-up studies makes it impossible to draw any firm conclusions about the future abstinence after release from detention.

In light of the limitations indicated above, it would be interesting to continue to apply the contingency management program in conjunction with other treatment programs, as proposed by Fernández-Moreno et al. (2022). It is recommended that future studies use larger and more heterogenous samples of juvenile offenders in different detention regimes, and include a follow-up period after the detention period to evaluate the impact of the treatment with a social context. Future research should also consider other variables which may have conditioned the results of the present study, particularly cognitive variables which may have been influenced by participation in the contingency management program.

In conclusion, contingency management programs, applied in conjunction with the necessary disciplinary regimes and procedures established in each institution, may increase the effectiveness of other intervention programs in reducing the prevalence of relapses in drug use among young offenders.

## Data availability statement

The datasets presented in this article are not readily available because in order to share the data with other interested parties, express authorization from the Agency for the Rehabilitation and Reintegration of Juvenile Offenders of the Community of Madrid is required. Requests to access the datasets should be directed to carlosbenedicto@ginso.org.

## Ethics statement

The studies involving humans were approved by Ethics Biosafety Committee of the Complutense University of Madrid. For each application of biological tests for the drug use detection, written informed consent was given to the participants. The legal representatives were informed and asked to consent to the performance of the relevant medical tests for the treatment of the different pathologies that occurred during the internment. The outcome of this study is the result of a retrospective analysis of the medical tests performed. The studies were conducted in accordance with the local legislation and institutional requirements. Written informed consent for participation in this study was provided by the participants' legal guardians/next of kin.

## Author contributions

ÁF-M: Conceptualization, Data curation, Investigation, Methodology, Writing – original draft, Writing – review & editing. DR: Formal analysis, Investigation, Methodology, Supervision, Writing – original draft, Writing – review & editing, Conceptualization. RM-F: Formal analysis, Methodology, Supervision, Writing – original draft, Writing – review & editing.
